# The association between social support and antenatal depressive and anxiety symptoms among Australian women

**DOI:** 10.1186/s12884-021-04188-4

**Published:** 2021-10-22

**Authors:** Asres Bedaso, Jon Adams, Wenbo Peng, David Sibbritt

**Affiliations:** 1 Hawassa University, College of Medicine and Health Sciences, School of Nursing, Hawassa, Ethiopia; 2 Australian Centre for Public and Population Health Research, School of Public Health, Faculty of Health, University of Technology Sydney, Ultimo, NSW Australia

**Keywords:** Depressive symptoms, Anxiety symptoms, Social support, Emotional support, Affectionate support, Tangible support, Women, Pregnancy

## Abstract

**Background:**

Antenatal depression and antenatal anxiety adversely affect several obstetric and foetal outcomes, and increase the rate of postnatal mental illness. Thus, to tackle these challenges the need for social support during pregnancy is vital. However, an extensive literature search failed to show a published study on the relationship between domains of social support and antenatal depressive, as well as antenatal anxiety symptoms in Australia. This study examined the association between domains of social support and antenatal depressive and anxiety symptoms among Australian women.

**Methods:**

The current study used data obtained from the 1973–78 cohort of the Australian Longitudinal Study on Women’s Health (ALSWH), focusing upon women who reported being pregnant (*n* = 493). Depression and anxiety were assessed using the 10 item Center for Epidemiological Studies Depression (CES-D-10) scale, and the 9-item Goldberg Anxiety and Depression scale (GADS) respectively. The 19 item-Medical Outcomes Study Social Support index (MOSS) was used to assess social support. A logistic regression model was used to examine the associations between domains of social support and antenatal depressive and anxiety symptoms after adjusting for potential confounders.

**Result:**

The current study found 24.7 and 20.9% of pregnant women screened positive for depressive and anxiety symptoms respectively. After adjusting for potential confounders, our study found that the odds of antenatal depressive symptoms was about four and threefold higher among pregnant women who reported low emotional/informational support (AOR = 4.75; 95% CI: 1.45, 15.66; *p* = 0.010) and low social support (overall support) (AOR = 3.26; 95%CI: 1.05, 10.10, *p* = 0.040) respectively compared with their counterpart. In addition, the odds of antenatal anxiety symptoms was seven times higher among pregnant women who reported low affectionate support/positive social interaction (AOR = 7.43; 95%CI: 1.75, 31.55; *p* = 0.006).

**Conclusion:**

A considerable proportion of pregnant Australian women had depressive symptoms and/or anxiety symptoms, which poses serious health concerns. Low emotional/informational support and low affectionate support/positive social interaction have a significant association with antenatal depressive and anxiety symptoms respectively. As such, targeted screening of expectant women for social support is essential.

**Supplementary Information:**

The online version contains supplementary material available at 10.1186/s12884-021-04188-4.

## Background

Pregnancy is a period that brings physiological and psychosocial changes in women, which increases the risk for the occurrence of mental illness [1, 2]. Depression and anxiety are among the most common mental illness occurring during pregnancy [3, 4].

Depression is characterized by symptoms of depressed mood, or loss of interest, low self-esteem, feelings of worthlessness, loss of appetite, feelings of fatigue, and poor concentration [5]. A previous meta-analysis conducted in developed countries reported a 7.4, 12.8 and 12% pooled prevalence of antenatal depression during the 1st, 2nd and 3rd trimester, respectively [6]. Also, a 25.3% pooled prevalence of antenatal depression was reported by a meta-analysis conducted in low and middle-income countries (LMICs) [7]. An estimated prevalence of antenatal depression reported by studies conducted in Australian ranges, 6–6.2% [8, 9], 7% [10] and 16.9% [11].

Antenatal anxiety is defined as excess concerns about the pregnancy, childbirth, health of the infant and future parenting roles [12]. Based on a report of global level meta-analysis the pooled prevalence of antenatal anxiety estimated a 34.4% pooled prevalence in LMICs and 19.4% in high-income countries [13]. Studies conducted in Australia revealed that the prevalence of antenatal anxiety ranges between 14 and 59% [14–19].

The risk factors for antenatal anxiety include previous pregnancy loss [20–22], stress [23], abuse during pregnancy [23–25], history of mental illness [23, 25–27], smoking/substance abuse [21, 23, 28, 29], drinking alcohol [28], antenatal depression, food insecurity, unplanned pregnancy [30], low social support, and poor quality relationship with a partner [31]. The risk factors for antenatal depression include anxiety, low social support, major life events, low income, history of abuse [8, 11, 32], domestic violence [32], unplanned pregnancy, history of any mental illness, and stress [33, 34].

Antenatal depression and anxiety adversely affect several obstetric and foetal outcomes and cause an increased rate of pregnancy complications and postnatal mental health problems [35–38]. Untreated antenatal anxiety and depression may lead to postnatal depression for the mother which may also result in an impaired interaction with her infant [39–41]. Thus, to tackle these challenges the need for social support during pregnancy is vital [42].

Social support is defined as the provision of financial, instrumental, emotional, and psychological support for somebody by a social network of family members, friends, and community members [43]. It strengthens social relationships and promotes health and well-being for a successful pregnancy [44]. However, the relationship between the specific domains of social support and antenatal depression, as well as antenatal anxiety symptoms remains understudied in Australia and globally. In addition, within the available evidence, there is a knowledge gap and reported inconsistencies regarding the association between specific domains of social support and mental health problems among pregnant women. Understanding the relationship between specific domains of social support and antenatal depression, as well as antenatal anxiety can assist in the process of establishing a specific type of community-based social support program to enhance the mental wellbeing of pregnant women.

Therefore, to address the abovementioned gaps in the current literature this study examined the association between domains of social support and antenatal anxiety and antenatal depressive symptoms amongst Australian women.

## Methods

### Study design, population and sampling

The current study employed a community based cross-sectional study design and reported per the guideline of the STROBE checklist (Additional file 1). This study analyses data from the 1973–78 cohort of the Australian Longitudinal Study on Women’s Health (ALSWH) [45, 46]. ALSWH is a community-based longitudinal study focusing on the health and well-being of Australian women. Over 40,000 women were recruited to participate in 1996 (baseline) in three cohorts (1973–78, 1946–51 and 1921–26). Participants were selected randomly via the national health insurance database and completed mailed surveys every 3 years on average. From the 8010 women who completed Survey 6 of the 1973–78 cohort in 2012, those who reported being pregnant (*n* = 493) (age between 34 and 39 years) were included in the current analyses [47]. Additional Information about the ALSWH can be found on the project website (www.alswh.org.au) and elsewhere [46].

### Outcome and exposure variables

Antenatal depressive and anxiety symptoms were the outcome variables. The depressive symptom was assessed using the 10-item Center for Epidemiological Studies Depression (CES-D-10, possible range 0–30) scale. A cut-off point > 10 out of 30 indicates the presence of depressive symptoms [48]. It has been used to examine depressive symptoms during pregnancy with good reliability (α = 0.79) and validity (sensitivity: 97%; specificity: 84%) [49–53]. The anxiety symptoms were assessed using the anxiety subscale of the Goldberg Anxiety and Depression scale (GADS) (yes/no, a possible range 0–9). GADS anxiety score of > 6, indicates the presence of anxiety symptoms and has good reliability (α = 0.77) [54] and validity (sensitivity: 83.1%, specificity: 81.8%). The GADS has been used with pregnant women in Australia [55].

The exposure variable for the current study was social support. The Medical Outcomes Study Social Support index (MOSS) was used to examine the functional support provided, with good reliability (α = 0.97) and validity [56, 57] and used among Australian women [58]. MOSS has an overall index of 19 items and four functional support subscales: emotional/informational support (8 items), tangible support (4 items), and affectionate/positive social interaction (7 items). Emotional/informational support is the expression of positive affect, being empathic, and providing advice or information which can provide a solution to a problem. Affectionate support/positive social interaction is the expression of love, affection and availability of others to share entertaining activities with an individual. Tangible/instrumental support is the provision of material or financial assistance. Each of the 19 items has a 5-point Likert response (ranging from: ‘none of the time’= 1 to ‘all of the time’= 5) assessing the availability of support. Overall score and each domain of social support were categorized into high (“all of the time” and “most of the time”) and low (“a little of the time/none” and “some of the time”) social support [56].

### Potential confounders and instruments used

Our study adjusted the potential confounders identified from previous studies and available in our dataset. The confounders were categorized into socio-demographic, behavioural and psychological, and obstetric factors. The socio-demographic confounders included; age, residence, marital status, highest educational qualification, and able to manage on available income [8, 11, 32]. Behavioural and psychological factors included; ever being in a violent relationship with a partner (yes/no) [31], substance use (current tobacco smoking, alcohol use, and ever used illicit drug) [21, 23, 28, 29], history of mental illness (history of depression in the past 3 years, and history of anxiety in the past three years) [33, 34], stress [23], and optimism. Finally, the obstetric confounding factors included; gestational age (months), GP use in the last 12 months, pre-term history [33, 34], BMI [30], and general health condition from their own perspective.

Study participants were requested to specify their marital status as either “married,” “never married,” “de facto,” “separated,” “divorced,” or “widowed.” For the current analysis, groups were re-categorized into either “partnered” (married or de facto) or “non-partnered” (single, divorced, separated, or widowed). Postcode of residence was used to categorise respondents as living in either “major cities of Australia”, “inner regional Australia”, “outer regional Australia” or “remote or very remote Australia” [59]. Income stress was measured via how respondents reported ability to manage on available income, with response options: “impossible”, “difficult all of the time”, “difficult some of the time”, “not too bad”, or “easy”. For the purposes of analyses, these options were collapsed into 2 categories, “impossible or difficult all or some of the time” and “not too bad or easy”.

The level of stress in the last 12 months among study participants was assessed using the Perceived Stress Questionnaire, which has been developed and validated for the ALSWH study [60]. The tool examined the level of perceived stress in specific areas of life, including study, relationships and own health. An overall mean stress score was determined, which ranges from 0 (no stress) to 4 (extreme stress). The Perceived Stress Questionnaire has good internal reliability (α = 0.75) [61, 62]. Also, the Life-Orientation Test-Revised (LOT-R) was used to examine optimism (a scale of 0–24).

The consumption of alcohol among study participants was assessed using the National Health and Medical Research Council (NHMRC) guidelines and categorized as: low-risk drinker; non-drinker; rarely drinks; risky/ high-risk drinker [63]. Due to the small number of responses, the consumption of alcohol was then re-categorized as being either “low risk-drinker” (non-drinker low-risk drinker/rarely drinks) or a “high-risk drinker” (risky/high-risk drinker) of alcohol. Also, based on their response to a question asking their current status of cigarette smoking, participants were categorized as being either a “non-smoker” or a “current smoker” of cigarettes. Study participants were asked if they used any of the following illicit drugs in the past 12-month; Marijuana; Amphetamines; LSD; Hallucinogens; Tranquillizers; Cocaine; Ecstasy/designer drugs; Inhalants; Heroin; Barbiturates; and Steroids. Based on their responses, the women were classified as being either a “non-user” or a “user” of an illicit drug.

Pregnancy body mass index (BMI) was assessed using self-reported weight during pregnancy (kg)/height (m) ^2^, and classified according to the WHO’s classification, underweight (< 18.5 kg/m^2^), normal weight (BMI 18.5–24.99 kg/m^2^), overweight (BMI of 25–29.9 kg/m^2^) and obese (BMI > 30 kg/m^2^) [64].

### Data analysis

Data were analysed using SPSS version 22. Chi-square tests and independent sample t-test analysis were performed to test for crude associations between the outcome variables (antenatal anxiety and depressive symptoms) and the confounding variables. Exposure variables include low emotional/instrumental support, low affectionate support/positive social interaction and low tangible support. The prevalence of antenatal anxiety and antenatal depressive symptoms was calculated for each of the independent variables.

During the bivariate analyses, variables with *p* < 0.25 were entered into a multiple logistic regression model and adjusted for confounder. In the final model, the strength of association between the outcome variables and domains of social support was measured by adjusted odds ratios (AOR) with corresponding 95% confidence intervals. The significance level was set at *p* < 0.05. The final model was assessed using the Hosmer and Lemeshow goodness of fit test [65].

A sensitivity analysis was conducted to estimate the E-values to assess the effect of unmeasured confounding [66]. The E-value is the minimum strength of association on the odds ratio estimate that an unmeasured confounder possibly will require to have with both the exposure and outcome to negate the reported associations based on measured confounders [67, 68].

## Result

### Demographic characteristics of participants

Table 1 describes the demographic characteristics of the study participants according to their risk of depression and anxiety as examined by the CESD-10 and GAD scale respectively. The prevalence of antenatal depression and anxiety in the current study was found to be 24.7% (95% CI: 21.2, 28.9), and 20.9% (95% CI: 17.5, 24.8) respectively. Also, 13.6% of pregnant women had comorbid depressive and anxiety symptoms. Of the total study participants, 11.8 and 6.5% had a history of depression and anxiety in the past 3 years respectively.

**Table 1:** The associations between sociodemographic characteristics of pregnant women and antenatal depression and anxiety (*n* = 493)

	Antenatal Depression			***p***-value	Antenatal Anxiety			
Demographic characteristics	No (***n*** = 365)	Yes (***n*** = 122)	Total (n = 493)		No (***n*** = 390)	Yes (***n*** = 103)	Total (n = 49)	p-value
	n (%)	n (%)	n (%)		n (%)	n (%)	n (%)	
**Residence**								
Major cities	220 (63.8)	64 (54.7)	284(61.4)	0.210	230 (62.3)	56(56.6)	286(61.1)	0.514
Inner regional	69 (20)	31 (26.5)	100(21.6)		80 (21.7)	23 (23.2)	103(22)	
Outer regional/remote/very remote	56 (16.2)	22 (18.8)	78(17)		59 (16)	20 (20.2)	79(16.9)	
**Highest qualification**								
University	246 (67.4)	71 (58.7)	317(63.9)	0.272	257 (66.2)	62 (60.2)	319(65)	0.517
Certificate/diploma or trade/apprenticeship	80 (21.9)	31 (25.6)	111(22.4)		85 (21.9)	27 (26.2)	112(22.8)	
School only	39 (10.7)	29 (15.7)	68(13.7)		46 (11.9)	14 (13.6)	60(12.2)	
**Marital status**								
Partnered	352 (96.4)	111 (91)	463(95)	0.016	370 (95.1)	98 (95.1)	468(95.1)	0.990
non-partnered	13 (3.6)	11 (9)	12(5)		19 (4.9)	5 (4.9)	24(4.9)	
**Able to manage on income available**								
Impossible/Difficult all of the time	18 (4.9)	25 (20.7)	43(8.8)	< 0.001	23 (5.9)	20 (19.6)	43(8.8)	< 0.001
Difficult some of the time	82 (22.5)	32 (26.4)	114(23.5)		89 (22.9)	29 (28.4)	118(24)	
Not too bad/It is easy	265 (72.6)	64 (52.9)	329(67.7)		277 (71.2)	53 (52)	330(67.2)	
	**Mean (SD)**	**Mean (SD)**		**p-value**	**Mean (SD)**	**Mean (SD)**		**p-value**
**Age**	35.78(1.39)	36.1 (1.45)	35.8(±1.40)	0.029	35.86 (1.42)	35.85 (1.39)	35.8(±1.4)	0.977

The mean (±SD) age of the participants was 36.3 (standard deviation [SD] =1.42) and the majority of participants (95.1%) were married/De facto, while (65%) achieved a university degree. The majority of women (61.1%) lived in major cities and there was a significant mean age difference between pregnant women with and without depressive symptoms and it was higher among pregnant women with depressive symptoms (*p* = 0.029). There was a significant relationship between the marital situation and depression status of participants, those with depressive symptoms were more likely to be non-partnered (*p* < 0.016).

### Behavioural, psychological and obstetric characteristics of participants

The behavioural, psychological, and obstetric characteristics of the study participants are presented in Table 2. The majority of the women (42.1%) were in the last trimester of their pregnancy, while 37.5 and 20.5% were in the second and first trimester respectively. There was a significant association between women’s use of GP in the last 12-month and antenatal depressive (*p* = 0.025) and antenatal anxiety symptoms (*p* < 0.001). From the total study participants, 38.4% of them reported > 5 times General Practitioner (GP) use in the last 12 months. In terms of birth history, 14.3, 7 and 4.7% of participants had Cesarean section (C/S), preterm and low birth weight history respectively. Based on WHO BMI classification, most pregnant women (54.4%) had normal weight and 2.7% were underweight. Also, there was a significant association between BMI and antenatal depressive symptoms among study participants, and those with depressive symptoms were more likely to be obese (*p* = 0.034).

**Table 2:** The association between behavioral, psychological and obstetric characteristics of pregnant women and antenatal depression and anxiety (n = 493)

	Antenatal Depression			p-value	Antenatal Anxiety			p-value
Variables	No (n = 365)	Yes (n = 122)	Total (n = 493)		No (n = 390)	Yes (n = 103)	Total (n = 493)	
	n (%)	n (%)	n (%)		n (%)	n (%)	n (%)	
**Pregnancy months**								
< 3 month	74 (20.3)	25 (20.5)	99(20.3)	0.950	81 (20.8)	20 (19.4)	101(20.4)	0.746
3–6 month	140 (38.4)	45 (36.9)	185(38)		143 (36.7)	42 (40.8)	185(37.5)	
> 6 month	151 (41.4)	52 (42.6)	203(41.7)		166 (42.6)	42 (39.8)	208(42.1)	
**GP use in the last 12 month**								
1–2 times	108 (29.7)	22 (18)	130(26.7)	0.025	116 (29.8)	16 (15.5)	132(26.8)	< 0.001
3–4 times	125 (34.3)	43 (35)	168(34.6)		137 (35.2)	32 (31.1)	169(34.3)	
> 5 times	131 (36)	57(46.7)	188(38.7)		136 (35)	55 (53.4)	191(38.9)	
**History of C/S**								
Yes	49 (13.5)	18 (14.9)	67(13.9)	0.710	55 (14.2)	15 (14.6)	70(14.3)	0.935
No	313 (86.5)	103 (21.3)	416(86.1)		331 (85.8)	88 (85.4)	419(85.7)	
**Preterm history**								
Yes	24 (6.7)	10 (8.3)	34(7)	0.553	30 (7.8)	4 (3.9)	34(7)	0.173
No	336 (93.3)	111 (91.7)	447(93)		355 (92.2)	98 (96.1)	453(93)	
**Low birth weight history**								
Yes	16 (4.4)	7 (5.7)	23(4.8)	0.558	19 (4.9)	4 (3.9)	23(4.7)	0.650
No	345 (95.6)	115 (94.3)	460(95.2)		367 (95.1)	99 (96.1)	466(95.3)	
**Body Mass Index (BMI)**								
Acceptable/Underweight(< 25)	218 (60.7)	58 (47.5)	276(57.4)	0.034	224 (58.2)	54 (52.9)	278(57.1)	0.603
Overweight (25–30)	87 (24.2)	37 (30.3)	124(25.8)		98 (25.5)	28 (27.5)	126(25.9)	
Obese (> = 30)	54 (15)	27 (22.1)	81(16.8)		63 (16.3)	20 (19.6)	83(17)	
**General Health condition from their own perspective**								
Excellent/Very good	280 (76.7)	68 (55.7)	348(71.5)	< 0.001	297 (76.2)	55 (53.4)	352(71.4)	< 0.001
Good	76 (20.8)	46 (37.7)	122(25)		86 (22.1)	38 (36.9)	124(25.2)	
Fair/Poor	9 (2.5)	8 (6.6)	17(3.5)		7 (1.8)	10 (9.7)	17(34.4)	
**Ever been in a violent relationship with partner**								
Yes	22 (6.1%)	30 (71.4)	52(11)	< 0.001	29 (7.5)	23 (23)	52(10.9)	< 0.001
No	337 (93.1)	85 (25.2)	422(89)		352 (91.2)	75 (75)	427(89.1)	
**Current tobacco smoking**								
Yes	13 (3.6)	9 (7.4)	22(4.5)	0.079	16 (4.1)	6 (5.8)	22(4.5)	0.450
No	352 (96.4)	113 (92.6)	465(95.5)		374 (95.9)	97 (94.2)	471(95.5)	
**Alcohol use**								
Low/High risk drinker	261 (71.7)	79 (65.3)	340(70)	0.182	276 (71)	68 (66.7)	344(70)	0.400
Non-drinker	103 (28.3)	42 (34.7)	145(30)		113 (29)	34 (33.3)	147(30)	
**Ever used illicit drugs**								
Yes	228 (65.2)	88 (72.1)	316(65)	0.053	244 (62.6)	74 (71.8)	318(64.5)	0.081
No	137 (37.5)	34 (27.9)	171(35)		146 (37.4)	29 (28.2)	175(35.5)	
**Current Anxiety (GAD)**								
Yes (>= 6)	36 (9.9)	67 (54.9)	103(21.2)	< 0.001	_	_		
No (< 6)	328 (90.1)	55 (45.1)	383(78.8)		_	_		
**Current Depression (CES-D 10)**								
Yes (>= 8)	_	_			55(14.3)	67(65)	122(25)	< 0.001
No (< 8)	_	_			329(85.7)	36(35)	365(75)	
**History depression in the past 3 years (Previous Mental health)**								
Yes	27 (7.5)	30 (25)	57(11.9)	< 0.001	31 (8.1)	27 (26.5)	58(12)	< 0.001
No	332 (92.5)	90 (75)	422(88.1)		352 (91.9)	75 (73.2)	427(88)	
**History anxiety in the past 3 years (Previous Mental health)**								
Yes	14 (3.9)	18 (15)	32(6.7)	< 0.001	19 (5)	13 (12.7)	58(12)	0.005
No	345 (96.1)	102 (85)	447(93.3)		364 (85)	89 (87.3)	427(88)	
**Mean Stress score**								
≤0.27	86 (23.6)	2 (1.7)	88(18.2)	< 0.001	84 (21.6)	4 (3.9)	88(18)	< 0.001
0.28–0.56	162 (44.5)	29 (24)	191(39.4)		174 (44.8)	21 (20.6)	195(39.8)	
0.57–0.82	68 (18.7)	31 (25.6)	99(20.4)		73 (18.8)	26 (25.5)	99(20.2)	
0.83–4	48 (13.2)	59 (48.8)	107(22)		57 (14.7)	51 (50)	108(22)	
Mean (+SD)	0.61(+ 0.43)							
**LOT-R Optimism**								
0–14.99	59 (16.2)	57 (47.1)	116(23.9)	< 0.001	72 (18.5)	45 (43.7)	117(23.8)	< 0.001
15–17.99	88 (24.1)	28 (23.1)	116(23.9)		96 (24.7)	23 (22.3)	119(24.2)	
18–18.99	81 (22.2)	20 (16.5)	101(20.8)		86 (22.1)	17 (16.5)	103(20.9)	
19–24	137(37.5)	16(13.7)	153(31.5)		135(34.7)	18(17.5)	153(31.1)	
Mean (+SD)	16.85(+ 4.05)							

Note: p-value was based on chi-square test and t-test statistics, Abbreviation: C/S: Cesarean Section

The majority of participants (71.4%) reported excellent/very good general health condition. Participant’s general health condition status was significantly associated with their status of antenatal depressive (*p* < 0.001) and antenatal anxiety symptoms (p < 0.001). Participants with depressive as well as anxiety symptoms were more likely to have a fair/poor general health condition (*p* < 0.001).

Of the total study participants, 11% ever had a violent relationship with a partner and ever being in a violent relationship with a partner was significantly associated with antenatal anxiety (*p* < 0.001) and antenatal depressive symptoms (p < 0.001). Participants with depressive symptoms were more likely to have less optimism score (*p* < 0.001), and higher mean stress level (p < 0.001). Likewise, those with anxiety symptoms were more likely to have a higher mean stress level (*p* < 0.001) and less optimism score (p < 0.001). Compared to non-depressed participants, a higher proportion of women with depressive symptoms reported current anxiety symptoms (p < 0.001), a personal history of anxiety (p < 0.001), and depression in the past 3 years (p < 0.001). Also, compared to non-anxious participants, a higher percentage of pregnant women with anxiety symptoms reported current depressive symptoms (p < 0.001). Participants with current anxiety symptoms were more likely to have a personal history of depression (p < 0.001) and antenatal anxiety (p < 0.001) in the past 3 years.

### Associations between social support and depressive symptoms during pregnancy

The relationship between depressive symptoms and social support during pregnancy is presented in Table 3. After adjusting for potential confounders, the multiple logistic regression model found that the odds of antenatal depressive symptoms was fourfold higher among pregnant women who reported low emotional/informational support (AOR = 4.75; 95% CI: 1.45, 15.66; *p* = 0.010) compared with pregnant women who reported high emotional/informational support. Also, pregnant women who reported low social support (overall support) were three times more likely to be depressed (AOR: 3.26, 95% CI: 1.05, 10.10, *p* = 0.040) compared with their counterparts.

**Table 3:** Multiple logistic regression model, showing the association between social support and antenatal depressive symptoms among Australian women, after adjusting potential confounders (n = 493)

Variables	Antenatal Depression		AOR (95% CI)	p-value
	No (n = 365)	Yes (n = 122)		
	n (%)	n (%)		
Medical Outcomes Study Social Support Index				
Emotional/Informational support^†^				
High (All/Most of the time)	358 (98.1)	90 (73.8)	1	
Low (Some/none/little of the time)	7 (1.9)	32 (26.2)	4.75 (1.45, 15.66)	0.010
Affectionate/Positive social interaction^†^				
High (All/Most of the time)	361(98.9)	101(83.5)	1	
Low (Some/none/little of the time)	4(1.1)	20(16.5)	1.65 (0.38, 8.12)	0.53
Tangible Support^†^				
High (All/Most of the time)	341(93.4)	95(77.9)	1	
Low (Some/none/little of the time)	24(6.6)	27(22.1)	1.49 (0.53, 4.19)	0.44
Overall social support^¥^				
High (All/Most of the time)	356(97.5)	97(79.5)	1	
Low (Some/none/little of the time)	9(2.5)	25(20.5)	3.26 (1.05, 10.10)	0.040

^**†**^Model is adjusted for: marital status, age, GP use, BMI, general health condition from their own perspective, ever been in a violent relationship with partner, ever used illicit drug, current Tobacco smoking, Alcohol use, current anxiety symptoms, history of depression in the past 3 years, history of anxiety in the past 3 years, mean stress score, and optimism. Hosmer and Lemeshow Test (Chi-square = 4.6, df = 8, *p* = 0.799)

^¥^Model is adjusted for: marital status, age, GP use, BMI, general health condition from their own perspective, ever been in a violent relationship with partner, ever used illicit drug, current Tobacco smoking, Alcohol use, current anxiety symptoms, history of depression in the past 3 years, history of anxiety in the past 3 years, mean stress score, and optimism. Hosmer and Lemeshow Test (Chi-square = 1.839, df = 8, *p* = 0.986)

### Associations between social support and anxiety symptoms during pregnancy

Table 4 shows the association between anxiety symptoms and low social support during pregnancy. After adjusting for potential confounders, the multiple logistic regression model found that the odds of antenatal anxiety symptoms was seven times higher among pregnant women who reported low affectionate support/positive social interaction (AOR = 7.43; 95% CI: 1.75, 31.55; *p* = 0.006) compared with pregnant women who reported high affectionate support/positive social interaction.

**Table 4:** Multiple logistic regression model, showing the association between social support and antenatal anxiety symptoms among Australian women, after adjusting potential confounders (n = 493)

Variables	Antenatal Anxiety		AOR (95% CI)	p-value
	No (n = 390)	Yes (n = 103)		
	n (%)	n (%)		
Medical Outcomes Study Social Support Index				
Emotional/Informational support^†^				
High (All/Most of the time)	374(96.1)	79(76.7)	1	
Low (Some/none/little of the time)	15(3.9)	24(23.3)	1.12 (0.36, 3.47)	0.840
Affectionate/Positive social interaction^†^				
High (All/Most of the time)	384 (98.7)	83 (81.4)	1	
Low (Some/none/little of the time)	5 (1.3)	19 (18.6)	7.43 (1.75, 31.55)	0.006
Tangible Support^†^				
High (All/Most of the time)	356(91.8)	83(80.6)	1	
Low (Some/none/little of the time)	32(8.2)	20(19.4)	0.88 (0.32, 2.45)	0.811
Overall social support^¥^				
High (All/Most of the time)	375(96.4)	82(79.6)	1	
Low (Some/none/little of the time)	14(3.6)	21(20.4)	2.55 (0.92, 7.06)	0.071

^**†**^Model is adjusted for: marital status, ability to manage on available income, GP use, general health condition from their own perspective, ever been in a violent relationship with partner, ever used illicit drug, preterm history, current depressive symptoms, history of depression in the past 3 years, history of anxiety in the past 3 years, mean stress score, and optimism. Hosmer and Lemeshow Test (Chi-square = 5.627, df = 8, *p* = 0.689)

^¥^Model is adjusted for: marital status, ability to manage on available income, GP use, general health condition from their own perspective, ever been in a violent relationship with partner, ever used illicit drug, preterm history, current depressive symptoms, history of depression in the past 3 years, history of anxiety in the past 3 years, mean stress score, and optimism. Hosmer and Lemeshow Test (Chi-square = 5.238, df = 8, *p* = 0.732)

### Sensitivity analysis

We ran a sensitivity analysis to calculate the E-values to observe the effect of unmeasured confounders in the final adjusted model. It has been suggested that for measures of dichotomous outcomes, the respective E-values for point estimates and confidence interval can be obtained using the Odds ratio (OR) (outcome prevalence > 15%) and its corresponding 95%CI in the online E-value formula [67, 68]. Based on this assumption, statistical evidence from our E-values suggested that the odds ratio of the relationship between an unmeasured confounder and (i) low emotional support and antenatal depressive symptoms, (ii) low social support (overall support) and antenatal depressive symptoms and (iii) low affectionate support and antenatal anxiety symptoms would need to be at least 3.78, 3.01 (Additional file 2) and 4.89 (Additional file 3) respectively, for each association to negate the associations we found in the current study.

## Discussion

This study revealed several important findings in relation to the prevalence rate of anxiety and depression, as well as the relationship between low social support and mental health problems, including depressive and anxiety symptoms, during pregnancy among Australian women.

After adjusting for potential confounders, in the current study, low emotional/informational support was found to have a significant association with a higher odds of antenatal depressive symptoms. Likewise, the odds of depressive symptoms was three times higher among pregnant women who reported low social support (overall support) compared with their counterpart. Pregnant women with low emotional support/informational support may not have someone to confide in, obtain important information/advice from, or to help reduce the negative emotions associated with a distressing situation, and as a result, they might be exposed to stress and may later develop depression [69]. The positive relationship between low social support (overall support) and antenatal depressive symptoms was supported by other studies conducted in Australia and internationally. A facility-based cross-sectional study conducted in Turkey among pregnant women (*n* = 772) indicated that emotional and instrumental support, from the mother-in-law and general support from the husband had an inverse relation with antenatal depression [70]. Also, a study conducted in Australia on pregnant women (*n* = 367) emphasized that overall social support was negatively associated with antenatal depression [11]. Similarly, previous studies conducted in Ethiopia [71], Sweden [72], USA [73], Finland [74], and China [75] reported pregnant women who reported low social support were more likely to be depressed. However, none of the above-mentioned studies examined the relationship between specific domains of social support and antenatal depressive symptoms. On the contrary, a facility-based cross-sectional study conducted in Jordan (*n* = 218) reported that social support during pregnancy has no association with antenatal depression [76]. The possible reasons for this conflicting finding might be due to the variation in demographic characteristics of participants, and tools used to assess social support and adjustment of confounders. In the study conducted in Jordan, participants were those with age > 18 years and used the Duke Social Support and Stress Scale (DUSOCS) (12 items) which examines the amount of overall social support and number of supportive people [77]. However, in our study participants were between the age of 34–39 years old, used MOS-SSS (19 items) scale [56] to assess overall and domains of social support such as perceived emotional/informational support, tangible support, affectionate support, and positive social interaction.

Our study showed that pregnant women who reported low affectionate support/positive social interaction had a higher odds of anxiety symptoms compared to pregnant women who reported high affectionate support/positive social interaction. Pregnant women with low affectionate support/positive social interaction are less satisfied with family and poor in interacting with the social environment, and as a result, they might be exposed to loneliness, become less in emotional and stress coping ability and later become more anxious [69, 78]. However, we found insufficient statistical evidence for an association between low social support (overall support) and antenatal anxiety symptoms. Similar findings were reported from facility-based studies conducted in Greece (*n* = 165) [79] and Canada (*n* = 5271) [80], which stated low social support had no significant association with antenatal anxiety. On the contrary, studies conducted in the US [81], China [75, 82], and Germany [83], which all reported that low social support (overall support) had a significant association with antenatal anxiety symptoms. The possible reasons for this discrepancy might be due to the difference in demographic characteristics of participants, the instrument used to assess social support and adjustment of potential confounders. For instance, the study conducted in the US [81] used Turner Support Scale to assess only partner support given for pregnant women and adjusted only for confounders like maternal race/ethnicity, age, parity, education, pre-pregnancy BMI, and household income. However, our study used MOS-SSS (19 items) scale [56] to assess the overall and specific domains of social support (i.e., emotional support/informational support, affectionate support/positive interaction and tangible support) and adjusted for potential confounders from socio-demographic, behavioural and psychological characteristics of participants.

Almost 1 in 4 pregnant women in our study met the screening criteria for depressive symptoms. The finding is supported by several other studies conducted worldwide such as in Jamaica (25%) [84], Nigeria (24.9%) [85], Brazil (24.3%) [86], Vietnam (22.4%) [87] and South Africa (22%) [88]. However our finding is higher than a previous Australian national report (6%) [9] and other studies conducted in Australia, ranging from 7 to 17% [10, 11]. One possible reason that may account for the inconsistency regarding the prevalence of depressive symptoms during pregnancy between the current study and other studies conducted in Australia might be due to the use of instruments used to examine depression. That is, all studies conducted in Australia used EPDS (10 items) with a total score of > 13 considered a flag for possible depressive symptoms [89], however, the current study used the CES-D-10 screening tool, with a score > 10 suggestive of possible depressive symptoms [48].

Our study found that 1 in 5 pregnant women screened positive for anxiety symptoms. A comparable estimate of antenatal anxiety symptoms was reported from a study conducted in Canada (23%) [23], and a global level systematic review and meta-analysis, 18.2% (10 studies) for the 1st trimester and 19.1% (17 studies) for the 2nd trimester) [13]. However, a facility-based longitudinal cohort study conducted in Melbourne, Australia, reported a higher prevalence of antenatal anxiety (27.7%) [11]. The possible reason for the observed difference might be due to variation in demographic characteristics of participants, participant recruitment method, and instrument employed to screen antenatal anxiety symptoms. For example, in the study conducted in Melbourne, Australia, participants were between the age of 17–45 years, recruited participants from antenatal clinic attendees, followed longitudinal study design and used the Beck anxiety inventory scale (BAI-21 items) (a self-reported scale used to examine the level of physical and cognitive anxiety symptoms in the past week) [90] to screen antenatal anxiety, while in our study, participants were between the age of 34–39 years, recruited participants from a community, and used GADS (a score > 6 suggests the presence of anxiety symptoms) scale to screen antenatal anxiety symptoms.

Our study has some limitations that need to be considered when making inferences from our findings. First, the study relied on self-reported data from participants, which has the potential for recall bias to be introduced. Second, our findings are limited to pregnant women within the age range of 34–39 years and as such, our findings may not be generalizable to younger pregnant women. Third, as the data analysis was cross-sectional we cannot confirm the necessary time-based direction of events. Specifically, the presence of reverse causation between low social support and depressive and/or anxiety symptoms cannot be identified. Finally, the sample size of our study may have resulted in reduced statistical power and inflated *effect size* estimation. However, these limitations are countered by the fact that the study analyzed data collected from a nationally representative sample of pregnant women.

## Conclusion

A considerable proportion of pregnant Australian women had depressive and anxiety symptoms, which poses serious health concerns. Early screening of pregnant women for antenatal depressive and anxiety symptoms is important for the wellbeing of the mother and child. Low emotional/informational support and low affectionate support/positive social interaction have been identified as being significantly associated with antenatal depressive and anxiety symptoms respectively. As such we recommend that targeted screening of pregnant women for social support is important to prevent anxiety and/or depressive symptoms amongst pregnant women. Policy-makers and those working on maternity care need to consider the development of community-based social support programs to maintain the mental wellbeing of pregnant women. Finally, for future researchers, we recommend longitudinal studies with the view to examine the causative relationship between low social support and depressive and/or anxiety symptoms during pregnancy.

## Supplementary Information

**Additional file 1.** STROBE Statement—Checklist of items included in reports.

**Additional file 2.** Sensitivity analysis (E-values) for the association between low social support and the risk of antenatal depressive symptoms for the final adjusted model.

**Additional file 3.** Sensitivity analysis (E-values) for the association between low social support and the risk of antenatal anxiety symptoms for the final adjusted model.

## Data Availability

After request, all analyzed data will be available from the Australian Longitudinal Study on Women’s Health (ALSWH). https://www.alswh.org.au/.

## Abbreviations

ALSWH: Australian Longitudinal Study on Women’s Health
AOR: Adjusted Odds Ratio
BAI: Beck Anxiety Inventory
BMI: Body Mass Index
CESD-10: 10 item Center for Epidemiological Study Depression scale
C/S: Cesarean Section
DUSOCS: Duke Social Support and Stress Scale
EPDS: Edinburgh Postnatal Depression Scale
GADS: Goldberg Anxiety Depression Scale
GP: General Practitioner
LMICs: Low and Middle-Income Countries
LOT-R: Life Orientation Test-Revised
MOS-SSS-19: 19 item Medical Outcome Study Social Support Scale
STAI: State-Trait Anxiety Inventory
WHO: World Health Organization
