# Supplementary material for: The association between social support and antenatal depressive and anxiety symptoms among Australian women
Source: BMC Pregnancy Childbirth. 2021 Oct 22;21:708. doi: 10.1186/s12884-021-04188-4 (PMC8532351; doi:10.1186/s12884-021-04188-4)
Supplement: Supplementary file 3 — Additional file 3. Sensitivity analysis (E-values) for the association between low social support and the risk of antenatal anxiety symptoms for the final adjusted model. [file 12884_2021_4188_MOESM3_ESM.docx]

**Additional file 3**: Sensitivity analysis (E-values) for the association between low social support and the risk of antenatal anxiety symptoms for the final adjusted model.

|  |  |  | |
| --- | --- | --- | --- |
| Variables | **AOR (95% CI)** | **E-value** | |
|  |  | For point estimate | For CI |
| Low affectionate support/positive social interaction | 7.43 (1.75, 31.55) | 4.89 | 1.98 |

CI: Confidence Interval
